# Robust Lidar-Inertial Odometry with Ground Condition Perception and Optimization Algorithm for UGV

**DOI:** 10.3390/s22197424

**Published:** 2022-09-29

**Authors:** Zixu Zhao, Yucheng Zhang, Jinglin Shi, Long Long, Zaiwang Lu

**Affiliations:** 1 Institute of Computing Technology, Chinese Academy of Sciences, Beijing 100190, China; 2 University of Chinese Academy of Sciences, Beijing 100049, China

**Keywords:** lidar-inertial odometry, ground perception, state estimation, sensor fusion, UGV

## Abstract

Unmanned ground vehicles (UGVs) are making more and more progress in many application scenarios in recent years, such as exploring unknown wild terrain, working in precision agriculture and serving in emergency rescue. Due to the complex ground conditions and changeable surroundings of these unstructured environments, it is challenging for these UGVs to obtain robust and accurate state estimations by using sensor fusion odometry without prior perception and optimization for specific scenarios. In this paper, based on an error-state Kalman filter (ESKF) fusion model, we propose a robust lidar-inertial odometry with a novel ground condition perception and optimization algorithm specifically designed for UGVs. The probability distribution gained from the raw inertial measurement unit (IMU) measurements during a certain time period and the state estimation of ESKF were both utilized to evaluate the flatness of ground conditions in real-time; then, by analyzing the relationship between the current ground condition and the accuracy of the state estimation, the tightly coupled lidar-inertial odometry was dynamically optimized further by adjusting the related parameters of the processing algorithm of the lidar points to obtain robust and accurate ego-motion state estimations of UGVs. The method was validated in various types of environments with changeable ground conditions, and the robustness and accuracy are shown through the consistent accurate state estimation in different ground conditions compared with the state-of-art lidar-inertial odometry systems.

## 1. Introduction

With the rapid development of computer software technology and sensor hardware technology, the state estimation of UGVs utilizing various types of sensors, such as lidar, inertial measurement units (IMUs), stereo cameras and ultra-wide bands (UWBs), has been studied by many researchers during the past decade, and multi-sensor suites and different sensor fusion methods have been proposed that aim to improve the accuracy, efficiency and robustness of UGVs’ ego-motion state estimation [1]. Due to the physical characteristics of lidar-inertial odometry, it has a lower cost of computing resources and less degeneration in tough environments [2] compared with the other types of odometry, such as visual-inertial odometry. In addition, the fusion of lidar and IMU has been noticed and studied further for usage in UGVs in recent years. Furthermore, new types of lidar with a low cost and smaller size have also increasingly arisen in the market, such as solid-state lidar[3,4]. Based on the above analysis, our work mainly revolves around the improvements and optimizations of lidar-inertial odometry used in UGVs.

It is known that lidar odometry may degenerate in situations where large planar areas and few geometry features exist, so inertial information is always used to maintain robustness and boost the frequency of the output for the odometry system. Inertial information can provide valid pose transformation constraints and can be leveraged to compensate for the spinning motion of lidar. Furthermore, it can also be utilized for the coarse initialization of the lidar point cloud registration algorithm, such as iterative closest point (ICP) [5]. However, the perception of environmental elements of high-frequency inertial information gained from IMU is not taken seriously in many lidar-inertial odometry systems, and the information from lidar and IMU can only be fused through a manner that has fixed parameters and models as the environmental elements change. As a consequence, the adaptability to environments is limited and the accuracy of the state estimation of UGVs cannot stay consistent in changing environments. In this paper, we pay more attention to the potential capacity for the environmental perception of inertial information to advance the performance of lidar-inertial odometry. Since the ground surface is the only environmental element directly and physically contacted by UGVs and a large percentage of lidar points stay on the ground surface, the ground condition plays a key role in affecting the quality of a six degrees of freedom (DoF) ego-motion state estimation of lidar-inertial odometry. The fluctuation of the ground surface can cause changes in UGVs’ inertial information, which can be measured by IMU. As UGVs travel on the ground, with a perception of the ground condition in real-time, the parameters used in the lidar points processing algorithm can be adjusted specifically to adapt to the current environment and obtain more accurate state estimations of UGVs.

The error-state Kalman filter [6] is a common method that formulates the error state between the true state and nominal state as a probability distribution to fuse the state estimation evaluated by different sensors. Since ESKF is able to explain the process of sensor fusion from the perspective of probability, it is well-matched conceptually with the ground perception method proposed in the paper, which is also based on the probability distribution of IMU measurements, and the data flow from the ESKF framework can be reused in the process of the evaluation of the ground condition.

In this paper, a novel ground condition perception and optimization algorithm is presented that aims to enhance the performance of lidar-inertial odometry used in UGVs mainly in terms of robustness and accuracy. Several works were carried out based on the ESKF sensor fusion framework, and a new relationship between the IMU measurements and the accuracy of lidar odometry is founded and leveraged in order to bring the extra improvement in performance. The ground condition was firstly evaluated by the raw inertial information and then modified further according to the correction information from the reused data in ESKF. In addition, a specially designed vector was used to describe the ground conditions. Finally, the lidar points processing module responded to the current ground condition vector and the related parameters were optimized according to a formula. The formula was designed by analyzing the latent relationship between the current ground condition and the accuracy of lidar odometry. To the best of our knowledge, this is the first attempt to take the ground condition sensed by IMU into consideration to optimize the lidar-inertial odometry. The main contributions are introduced as follows.

- The novel ground condition perception and optimization algorithm is presented, and is composed of two methods. The first one is the method of ground condition perception using high-frequency inertial information and the data from the output of ESKF, and the second one is the method of the optimization of lidar points processing according to the output of the ground condition perception module.
- A framework of lidar-inertial odometry based on ESKF is proposed to estimate the state of UGVs’ poses, and the ground condition perception and optimization algorithm is well-embedded in this framework.
- Sufficient experiments were performed to validate the performance in terms of the accuracy and robustness of the system. Besides the open-source dataset, the environment with different ground conditions gathered by ourselves was also considered as part of the experimental dataset.

The rest of this paper is organized as follows. In Section 2, the related studies of lidar-inertial odometry are presented and analyzed. Section 3 talks about the framework and the main modules and algorithms of the odometry system. Section 4 introduces the experiments conducted in the open dataset and self-gathered dataset. Section 5 gives the conclusion and future work.

## 2. Related Work

As lidar-inertial odometry becomes increasingly irreplaceable in UGVs, there have been many theoretical studies about the fusion models of lidar and IMU and applied studies about the optimization methods designed for specific scenarios. In this part, based on the general architecture, we mainly focus on the works that aim toward the optimization of the accuracy, robustness and efficiency of lidar-inertial odometry, and the advantages and disadvantages of these methods are analyzed and stated.

The errors of sensor fusion odometry systems are generally thought to come from three sources: sensor noise, extrinsic calibration [7] and degenerated state estimation [8,9]. The sensor noise is always modeled as Gaussian noise or a similar variant, it is handled by the Kalman filter or pose graph in many optimization methods [10,11]. Regarding the extrinsic calibration of sensors, the parameters of the calibration are usually seen as parts of the robot state vector, and these vectors are optimized by various methods to satisfy the objective functions dynamically [12,13,14,15]. The degenerated phenomenon of lidar-inertial odometry in changing environments is hard to eliminate, and has attracted many researchers’ attention in recent years. An evaluation methodology of degeneracy was proposed by Ji Zhang [16]; it is used for an optimization-based state estimator. The degeneracy factor used to represent the degenerated directions is derived from the constraints brought by sensors’ measurements, and the final solution of the state estimation is optimized by trading-off the constraints in well-conditioned scenes and degenerated scenes. However, the threshold determining degeneration needs to be set manually, which is not suitable for changing environments. LINS [17] is the first work attempting to build a lidar-inertial odometry leveraging the effect of an iterated error-state Kalman filter, as the uncertainty of the robot state estimation grows with time [18]. A robocentric formulation is proposed to average the noisy measurements of different sensors; then, the degeneracy and divergency can be avoided. There is also a series of advanced lidar-inertial odometry [19,20,21,22] developed from the methodology of LINS. These methods show the advantage of the accuracy of pose estimation, but, as the idea of an averaging error is not suitable for severe degeneration, failure solutions may occur and the direction of degeneration cannot be determined in these methods. LIO-SAM [23] is the representative work that utilizes graph optimization methods to avoid the degeneration of lidar-inertial odometry; the state constraints generated in degenerated environments are seen as edges and vertices of the factor graph [24] with lower weights. The graph-based methods rely on the information matrix to measure the quality of state constraints. The drawback is that the information matrix may have singular values and cause a local optimum. These methods mentioned above tend to cope with degeneration by analyzing the uncertainty of the state estimation of lidar-inertial odometry (LIO) systems, but the capacity of the odometry to perceive and adapt to the environment in real-time is not paid enough attention; therefore, the performances in different environments may differ a lot.

As the ground occupies a large part of the lidar scan of the surrounding environment, it is an non-negligible environmental element for LIO systems used in UGV. Despite the general methods mentioned above, several works embedded with ground-optimized methods are proposed to preserve the qualities of state estimation, especially for UGVs [25,26,27,28,29]. LeGO-LOAM [25] is a loosely coupled lidar-inertial odometry that utilizes the technique of ground optimization in various terrain. The sensor noise from the ground is notified, and the solution to eliminate the noise is to refer to the ground separation of lidar point clouds and the two-step optimization method for state estimation: at each step, suitable features of the point cloud are chosen to calculate the corresponding components of the six-degrees-of-freedom poses of the UGV. This shows advantages in terms of efficiency and accuracy in the scenarios where stable ground features exist, but its accuracy may degrade in bumpy road conditions as the ground is not clear enough to be separated. HDL-Graph-SLAM [26] is a tightly coupled sensor fusion odometry system based on normal-distributions transform (NDT) [30] lidar scan matching and graph optimization. Lidar odometry, IMU measurements, GPS and loop-closing are regarded as the constraints of the poses of moving robots; furthermore, the ground-plane constraint is also added to correct the accumulated rotational error, which is hard to compensated for by loop closure on a plane. It assumes the presence of a unified plane in large indoor public environments, so the application scenarios are limited. These ground optimization methods are mostly designed for environments with a flat ground, and are not suitable for environments with changeable terrains.

Based on the ESKF, which handles the fusion of the measurements of lidar and IMU as the fusion of two probabilistic models, we decided to explore more relationships between lidar and IMU to further increase the accuracy and robustness of lidar-inertial odometry. In some popular and efficient pure lidar odometry systems that are based on the normal distributions of lidar points in voxel grids, such as SuMa [31] and LiTAMIN [32,33], the appropriate voxel size in different environments is pointed out to be a key factor influencing the accuracy of lidar odometry. In this paper, motivated by this mechanism and the idea of ground optimization, ground condition sensing was carried out by analyzing the inertial information, and the optimizing process was performed by adjusting the related parameters of the lidar points processing algorithm. As a consequence, the overall performance of the lidar-inertial odometry is improved.

## 3. Methodology

The following contents mainly introduce the architecture of the lidar-inertial odometry system and the key methods, including the ground condition perception algorithm, the lidar points processing optimization algorithm and the method used to embed these algorithms into the ESKF framework.

### 3.1. System Overview

**Figure 1:**
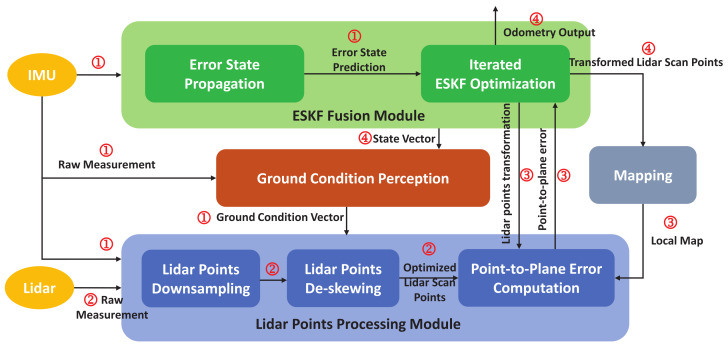
The overall architecture of the lidar-inertial odometry system proposed in this paper.

The overall architecture of our system and the pipeline of the dataflow are shown in Figure 1. The system is composed of four functional modules, including the ground condition perception module (Section 3.2), lidar points processing module (Section 3.3), mapping module (Section 3.4) and ESKF fusion module (Section 3.5). The pipeline of the dataflow is shown as the following procedure.

1. The raw measurements from IMU are fed into the ESKF fusion module to propagate the error state of UGV’s poses. At the same time, the inertial measurements are utilized in the ground condition perception module to calculate the ground condition vector. Furthermore, the inertial information is cached in the lidar points processing module for lidar points de-skewing.
2. The lidar scan points from the lidar are sent to the lidar points processing module for downsampling and de-skewing, and the parameters used in the points downsampling are determined by the ground condition vectors from the ground condition perception module. The optimized lidar scan points are produced during this procedure.
3. The optimized lidar points, the local map maintained by the mapping module and the lidar points transformation from the ESKF optimization are used when performing the point-to-plane error computation, and the parameters used in this procedure are also dynamically adjusted according to the ground condition vectors from the ground condition perception module.
4. ESKF optimization utilizes the point-to-plane error and the state propagated by IMU measurements to update the error state iteratively until the convergence is achieved. The final output of the odometry is gained from the state vector maintained by ESKF. At the same time, the state vector is used to transform the lidar scan points serving the mapping module; it can also be used in the ground condition perception for correction and calibration.

### 3.2. Ground Condition Perception

The raw measurements of IMU were utilized to evaluate the condition of the ground, which the moving UGV is currently in contact with. The raw inertial information *u* is composed of the angular velocity ω around three axes and the linear acceleration *a* on the three axes.

(1) $$u=\left[\omega \;a\right],\omega =\left[\omega_{x}\;\omega_{y}\;\;\omega_{z}\right],a=\left[a_{x}\;a_{y}\;a_{z}\right]$$

Suppose that the UGV moves straightforward, since the rise and fall of the ground may mainly cause the variance in angular velocity. Only the angular velocity ω is considered to measure the ground condition. In order to evaluate the flatness of the ground, the probability distribution of inertial rotational information from IMU measurements during a certain period of time was computed as the following procedure.

For a certain time period Δt between two consecutive lidar scans, suppose that there are *n* measurements of angular velocity. The average angular velocity $\bar{\omega }$ is computed. In order to eliminate the noise brought by IMU measurements and the effect of the random walk of IMU’s bias, the Gaussian white noise $n_{\omega }$ and the bias of angular velocity $b_{\omega }$ evaluated by the ESKF fusion module (Section 3.5) at the current time are utilized.

(2) $$\bar{\omega }=\frac{\sum_{i=1}^{n}\omega_{i}-b_{\omega_{i}}-n_{\omega_{i}}}{n}=\left[\bar{\omega_{x}},\;\;\bar{\omega_{y}},\;\;\bar{\omega_{z}}\right]$$

The covariance matrix $P_{\omega }$ of these measurements is also computed.

(3) $$\begin{array}{cc} \; & P_{\omega }=\left[\begin{array}{ccc} cov(\omega_{x},\omega_{x}) & cov(\omega_{x},\omega_{y}) & cov(\omega_{x},\omega_{z})\\ cov(\omega_{y},\omega_{x}) & cov(\omega_{y},\omega_{y}) & cov(\omega_{y},\omega_{z})\\ cov(\omega_{z},\omega_{x}) & cov(\omega_{z},\omega_{y}) & cov(\omega_{z},\omega_{z}) \end{array}\right]\\ \; & cov\left(X,Y\right)=\frac{\sum_{i=1}^{n}\left(X_{i}-\bar{X}\right)\left(Y_{i}-\bar{Y}\right)}{n} \end{array}$$

Then, the probability distribution of the measurements during the time period Δt can be obtained as $N(\bar{\omega },\;\;P_{\omega })$. However, since UGVs do not always move straightforwardly, the uncertainty of N is not caused entirely by the variance in the ground surface. In order to eliminate the effect of the active steering of UGVs, principle component analysis (PCA) was performed on the covariance matrix $P_{\omega }$.

(4) $$\begin{array}{cc} \; & P_{\omega }=Q\sum Q^{T}\\ \; & Q=\left[\begin{array}{ccc} v1 & v2 & v3 \end{array}\right],\;\sum =\left[\begin{array}{ccc} \lambda 1 & 0 & 0\\ 0 & \lambda 2 & 0\\ 0 & 0 & \lambda 3 \end{array}\right] \end{array}$$

∑ is the matrix composed of the eigen values, where λ1>λ2>λ3, and *Q* is the matrix composed of the eigen vectors of the corresponding eigen values. According to the theory of PCA, the eigen vector that has the largest eigen value is the main component of the covariance matrix. As for $P_{\omega }$, v1 represents the main direction where the orientation of IMU’s measurements varies. If the UGV begins to steer, λ1 will be much larger than λ2 and λ3; in order to remove the influence of steering and ensure that the uncertainty of probability distribution N is purely caused by the variance in the ground condition, $P_{\omega }$ and N were modified according to the following strategy.

(5) $$\begin{array}{cc} \; & P_{\omega }^{\prime }=Q\sum^{\prime }Q,\;\;N^{\prime }=N\left(\bar{\omega },\;\;P_{\omega }^{\prime }\right)\\ \; & \sum^{\prime }=\left[\begin{array}{ccc} {\lambda 1}^{\prime } & 0 & 0\\ 0 & \lambda 2 & 0\\ 0 & 0 & \lambda 3 \end{array}\right],\\ \; & {\lambda 1}^{\prime }=\left\{\begin{array}{c} (\lambda 2+\lambda 3)/2,if\lambda 1>>\lambda 2,\\ \lambda 1,else \end{array}\right. \end{array}$$

Inspired by the work carried out by LiTAMIN2 [33], which uses symmetric KL divergence to evaluate the difference in two distributions of point clouds, the KL-divergence was also utilized to compare two different distributions of IMU measurements in this paper. We formulated the difference between two series of IMU measurements gained in two consecutive time periods as the symmetric KL divergence of the two probability distributions $N_{1}\left(\omega_{1},P_{\omega_{1}}\right)$ and $N_{2}\left({\omega_{2},P}_{\omega_{2}}\right)$ transformed by Equation (5).

(6) $$\begin{array}{cc} Div\left(N_{1},\;N_{2}\right)= & {\left(\omega_{1}-\omega_{2}\right)}^{T}{\left(P_{\omega_{1}}+P_{\omega_{2}}\right)}^{-1}\left(\omega_{1}-\omega_{2}\right)+\\ \; & tr\left(P_{\omega_{1}}^{-1}P_{\omega_{2}}\right)+tr\left(P_{\omega_{2}}^{-1}P_{\omega_{1}}\right)-2d \end{array}$$

tr(A) represents the trace of matrix *A* and *d* denotes the dimension of ω. Since the $Div\left(N_{1},\;N_{2}\right)$ indicates the degree of the relative changes in the ground where the UGV travels during two consecutive time periods, it can be used as the signal to activate the optimization of parameters in the lidar points processing module, which will be introduced in detail in Section 3.3.1 and Section 3.3.3.

Since the KL divergence only describes the relative changes in the ground condition, the absolute value was addressed by computing the trace of the covariance matrix. The trace is an indicator of the level of the linear transformations contained in the matrix, and is a suitable factor to measure the flatness of the ground. In addition, the linear velocity of UGV may vary all of the time during the process of the evaluation of the ground condition. The results may be inconsistent under changeable linear velocities. To address this problem and obtain consistent results, the most recent linear velocity $v_{k-1}$ was taken from the output of the EKSF module; it can serve as the denominator of the trace of the covariance matrix $P_{\omega_{k}}$ to eliminate the effect of linear velocity. The ground condition vector ${GC}_{k}$ is finally given as follows, including the absolute value and the relative value. *k* represents the index of the lidar scan.

(7) $${GC}_{k}=\left(\frac{tr\left(P_{\omega_{k}}\right)}{v_{k-1}},\;\;Div\left(N_{k-1},\;N_{k}\right)\right)$$

### 3.3. Lidar Points Processing Module

#### 3.3.1. Lidar Points Downsampling

**Figure 2:**
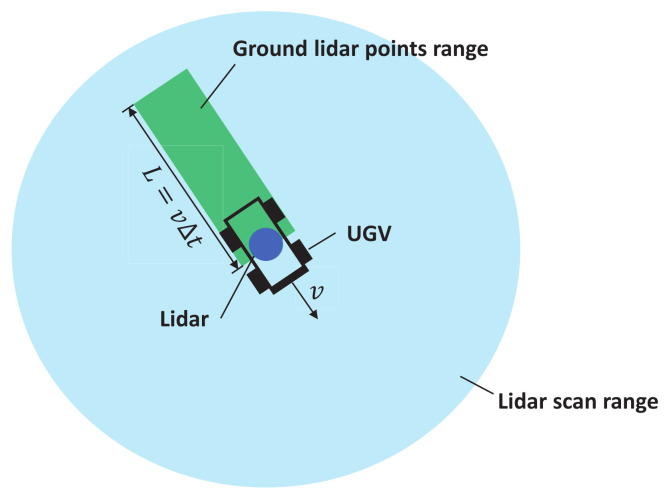
The range of the ground lidar points. *v* is the current velocity from the state vector evaluated by ESKF, Δt is the time period utilized to compute the distribution of IMU measurements and the width of the ground region is the width of the UGV.

In order to maintain the real-time performance of the odometry system, the lidar points were downsampled to improve the efficiency. The basic rate of downsampling was 1/4, which means that only one point was extracted for every four lidar points, which are temporally consecutive in the raw lidar scan. In this paper, due to the change in the ground condition, the downsampling rate of the lidar points lying on the ground should be dynamic; this is relevant to the ground condition vector computed in Equation (7). Firstly, the ground lidar points were segmented from the other points in a single lidar scan. Since the ground condition vector was computed from the inertial information of IMU, it can only represent the condition of the ground where the UGV directly contacts. Therefore, the segmented ground points are restricted to the region where UGV may have travelled on. The range of the ground lidar points is shown as the green rectangle region in Figure 2, and the height of points in the ground region should also be restricted to ±1 m. Then, the lidar points of the ground region were downsampled according to the ground condition vector ${GC}_{k}\left(tr\left(P_{\omega_{k}}\right),Div\left(N_{k-1},\;N_{k}\right)\right)$, and the downsampling rate $\;\alpha_{k}$ of the kth lidar scan was optimized according to the following strategy.

(8) $$\begin{array}{c} \alpha_{k}=\left\{\begin{array}{c} \alpha_{k-1},\mathrm{if}\;{GC}_{k}\left(1\right)<T_{activate},\\ {\left(\frac{1}{2}\right)}^{-\frac{2}{G_{max}}{GC}_{k}\left(0\right)+2},else \end{array}\right. \end{array}$$

Regarding the description in Section 3.2, ${GC}_{k}\left(1\right)=Div\left(N_{k-1},\;N_{k}\right)$ is regarded as the activating signal for the optimization of the downsampling rate and $T_{activate}$ is the threshold of the activating signal. When ${GC}_{k}\left(1\right)$ is relatively small, the downsampling rate is unchanged as the ground condition does not change too much; otherwise, it is adjusted according to ${GC}_{k}\left(0\right)=\frac{tr\left(P_{\omega k}\right)}{v_{k-1}}$. Suppose that the range of ${GC}_{k}\left(0\right)$ is $\left[0,G_{max}\right]$. The $G_{max}$ is an empirical value that is based on the engineering practice in real world environments. When ${GC}_{k}\left(0\right)=0$, which implies that the ground is a plane through the evaluation of ground perception, $\alpha_{k}$ takes the minimum value of 1/4, which is the same as the other region of the lidar scan, because the lidar points in the planar ground will not contribute too much to the state estimation of odometry. If ${GC}_{k}\left(0\right)$ is close to the upper bound, which means that the ground condition is rough enough, $\alpha_{k}$ takes the maximum value of 1, which represents that none of the points taken from the ground region are ignored. The details about the ground are saved so that more useful information from the ground points can be utilized for state estimation. We utilized an exponential function to represent the relationship between ${GC}_{k}\left(0\right)$ and $\alpha_{k}$. The changing rate of $\alpha_{k}$ is small when ${GC}_{k}\left(0\right)$ is close to zero, as the downsampling rate is not taken seriously when the ground is relatively flat.

#### 3.3.2. Lidar Points De-Skewing

A lidar scan consists of an accumulated set of points received during a short time period, but the lidar is generally considered to be static during the time period. In order to compensate for the small error led by the lidar moving, IMU measurements can be used to finely tune the positions of lidar points in the current lidar frame. Suppose that a lidar scan begins at $t_{0}$; the timestamp of the *j*th lidar point in the lidar scan is represented as $t_{j}$ and the original position of the point in the current lidar frame is $p_{j}$. The relative transformation per lidar point is composed of the rotation part $R_{t_{j}t_{0}}$ and the translation part $P_{t_{j}t_{0}}$. To simplify the representation, we used the form of the continuous function to express the computation of $R_{t_{j}t_{0}}$ and $P_{t_{j}t_{0}}$; in fact, they are accumulated by the discrete IMU measurements. $b_{\omega_{t}}$, $n_{\omega_{t}}$, $b_{a_{t}}$, $n_{a_{t}}$ and $v_{t}$ are from the state vector estimated by ESKF most recently in Section 3.5.

(9) $$\begin{array}{cc} \; & R_{t_{j}t_{0}}=EulerToRotationMatrix(\int_{t_{0}}^{t_{j}}(\omega_{t}-b_{\omega_{t}}-n_{\omega_{t}})dt)\;\\ \; & P_{t_{j}t_{0}}=\int_{t_{0}}^{t_{j}}v_{t}dt+\int \int_{t_{0}}^{t_{j}}(a_{t}-b_{a_{t}}-n_{a_{t}})dt \end{array}$$

Then, the tuned position $p_{j}^{\prime }$ per lidar point is computed.

(10) $$p_{j}^{\prime }=R_{t_{j}t_{0}}p_{j}+P_{t_{j}t_{0}}$$

#### 3.3.3. Point-to-Plane Error Computation

In order to compute the point-to-plane errors used in the iterated ESKF optimization (Section 3.5.3), a small plane $s_{i}$ was fitted by *n* map points; it consists of the center position ${{}^{W}q}_{i}$ in the world frame and normal vector $n_{i}$ representing the direction of the plane. These map points were founded by searching for the *n* points in the local map that are closest to the projection of the related point $p_{i}$ in the current lidar scan. The map construction is introduced in Section 3.4, and the point-to-plane error $E\left(p_{i},\;s_{i}\right)$ for each lidar point $p_{i}$ in the lidar frame and its corresponding plane $s_{i}$ in the map was computed as below.

(11) $$E\left(p_{i},\;s_{i}\right)={n_{i}({}^{W}R}_{I}\left({{}^{I}R}_{L}{{}^{L}p}_{i}+{{}^{I}P}_{L}\right)+{{}^{W}P}_{I}-{{}^{W}q}_{i})$$

${{}^{I}R}_{L}$ and ${{}^{I}P}_{L}$ are the transformation matrices from the lidar frame (denoted as *L*) to the IMU frame (denoted as *I*) that were calibrated previously, ${{}^{W}R}_{I}\;$ and ${{}^{W}P}_{I}$ represent the transformation matrices from the IMU frame to the world frame (denoted as *W*) that were taken from the state vector of IMU in the iterated ESKF optimization module and ${}^{L}p_{i}$ is the position of the ith lidar point in the current lidar frame.

**Figure 3:**
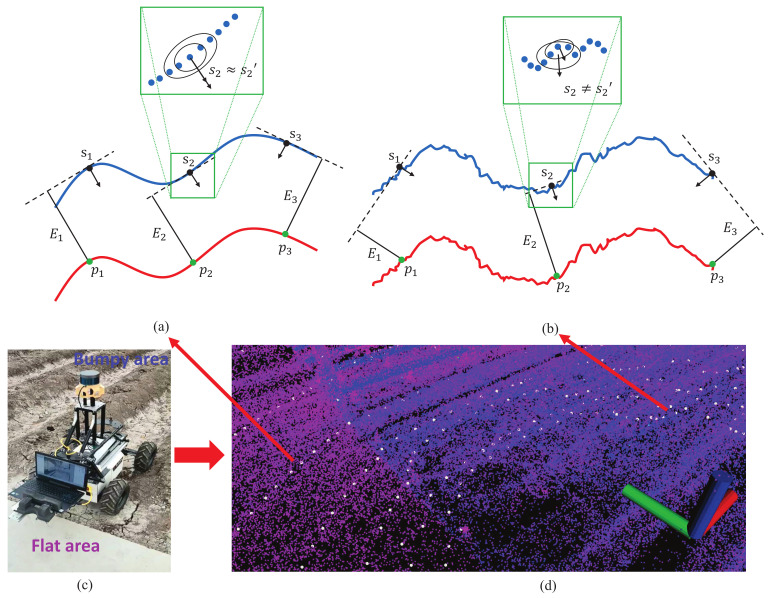
Point-to-plane error computation in different ground conditions. (**a**) The error computed in the ground of flat area. (**b**) The error computed in the ground of bumpy area. The blue line is the lidar points in the map, the red line is the lidar points in current lidar scan. $s_{i}$ is the local plane fitted by the nearest 5 map points from the point $p_{i}$, and ${s_{i}}^{\prime }$ is fitted by 3 points. $E_{i}$ is the associated point-to-plane error represented as the length of the related black line. The enlarged drawing at the top highlights the local plane fitted by the map points of different numbers. (**c**) The picture of real-world ground condition containing the planar area and bumpy area. (**d**) The related current lidar points and the surrounding local map points of picture (**c**), where the pink points and the blue points correspond to the map points in the flat area and bumpy area, respectively, and the white points are the lidar points in the current scan.

However, the number of considered map points used to fit the plane may have an influence on the result of the error computation. Figure 3 gives an example of the point-to-plane error computation in different ground conditions. In the flat area, the local plane $s_{i}$ is fitted correctly to reflect the local direction of the ground, and the normal vector of the fitted plane stays consistent as the number of considered map points changes ($s_{i}\approx {s_{i}}^{\prime }$). However, in the bumpy area where the ground surface is uneven, excessive points used to fit the local plane will result in different point-to-plane errors as the direction of the local plane varies too much with the number of considered map points ($s_{i}\neq {s_{i}}^{\prime }$). The excessive points cannot reflect the details of the local plane on an uneven ground surface, especially the direction represented by the normal vector. Consequently, the accuracy of the state estimation may be influenced. In order to cope with this problem, in the environments with changeable ground conditions, the points of the kth lidar scan used to compute point-to-plane errors need to be optimized using the following procedure. (i) The downsampling rate $\alpha_{k}$ of the points in the ground region is increased according to Equation (8) to keep the details of the ground. (ii) The number of map points $N_{k}$ used to fit the plane is decreased to obtain a more accurate local plane according to Equation (12). The basic number of considered map points in the flat area is 8, and it will decrease linearly as the flatness of the ground condition represented by ${GC}_{k}\left(0\right)$ grows, where the minimal number is restricted to 5. Round(x) is the rounding function.

(12) $$\begin{array}{c} N_{k}=\left\{\begin{array}{c} N_{k-1},if\;{GC}_{k}\left(1\right)<T_{activate},\\ Round(8-\frac{3{GC}_{k}\left(0\right)}{G_{max}}),else \end{array}\right. \end{array}$$

### 3.4. Mapping

The map maintained by the odometry system proposed in this paper is the collection of selected lidar points in the world frame during the whole process of the UGV’s state estimation. The map representation and construction methods were adopted from the work carried out by FAST-LIO2 [21]. Based on the incremental data structure of ikd-Tree used to manage the global map, the speed of the neighbor search of the nearest points is faster than the traditional kd-Tree, and the map updating (points insertion and deletion) also has a low time complexity of O(nlogn). These characteristics of ikd-Tree can guarantee the efficiency of the odometry system.

### 3.5. ESKF Fusion Module

The measurements from IMU and lidar were fused through a manner maintained by ESKF. Since ESKF has been well-studied in recent works of lidar-inertial odometry, only a brief process of ESKF is introduced in this section.

#### 3.5.1. Error State Representation

ESKF maintains the error state vector $\tilde{x}$, which is centric on the pose of IMU, and the error state was computed as below. ⊞/⊟ are the operator of addition and subtraction on the state vector.

(13) $$\tilde{x}=\;x⊟\hat{x}$$

*x* is the true state vector of IMU and composed of the elements shown below, and $\hat{x}$ is the predicted state vector propagated by IMU measurements.

(14) $$x=\left[{{}^{W}R}_{I}\;{{}^{W}P}_{I}\;{{}^{W}v}_{I}\;b_{\omega }\;b_{a}\right]$$

${{}^{W}R}_{I}$ and ${{}^{W}P}_{I}$ represent the rotation and translation from the IMU frame to the world frame, ${{}^{W}v}_{I}$ denotes the linear velocity of IMU and $b_{\omega }$ and ${\;b}_{a}$ represent the bias of IMU, which observes the distributions of random walk noise.

#### 3.5.2. State Propagation by IMU Measurements

Suppose that *k* denotes the index of lidar scans and *i* denotes the index of IMU measurements between two consecutive lidar scans, given the last state vector of IMU $x_{k-1}$; then, the predicted state vector at the time of current lidar ${\hat{x}}_{k}$ can be computed recursively by Equation (15) utilizing the inertial information between two consecutive lidar scans. ${\hat{x}}_{i}$ is the predicted state vector at the time of the ith IMU measurement, and the recursive formula $f({\hat{x}}_{i},u_{i},w_{i})$ of the predicted state vector is described below. $u_{i}$ is the IMU input and $w_{i}$ is the noise vector composed of IMU white noise and IMU bias noise.

(15) $$\begin{array}{cc} \; & {\hat{x}}_{i+1}=\;{\hat{x}}_{i}⊞\left(\Delta tf\left({\hat{x}}_{i},u_{i},w_{i}\right)\right)\\ \; & u_{i}=\left[\omega_{i}\;a_{i}\right],w_{i}=\left[n_{\omega_{i}}\;n_{a_{i}}\;n_{b_{\omega_{i}}}\;n_{b_{a_{i}}}\right]\\ \; & f\left({\hat{x}}_{i},u_{i},w_{i}\right)=\left[\begin{array}{c} \omega_{i}-{\hat{b}}_{\omega_{i}}-{\hat{n}}_{\omega_{i}}\\ \hat{{{}^{W}v}_{I_{i}}\;}\\ \begin{array}{c} \hat{{{}^{W}R}_{I_{i}}\;}\left(a_{i}-{\hat{b}}_{a_{i}}-{\hat{n}}_{a_{i}}\right)\\ n_{b_{\omega_{i}}\;}\\ n_{b_{a_{i}}\;} \end{array} \end{array}\right] \end{array}$$

Let ${\hat{P}}_{k}$ represent the covariance matrix measuring the uncertainty of the predicted state ${\hat{x}}_{k}$, starting from ${\hat{P}}_{k-1}$. ${\hat{P}}_{k}$ can be also propagated recursively using the following formula.

(16) $${\hat{P}}_{i+1}=F_{x_{i}}{\hat{P}}_{i}F_{x_{i}}^{T}+F_{w_{i}}Q_{i}F_{w_{i}}^{T}$$

The details on $F_{x_{i}}$, $F_{w_{i}}$ and $Q_{i}$ are introduced in the supplementary material of R${}^{2}$ LIVE [20]. $F_{x_{i}}$ and $F_{w_{i}}$ are the linearized propagation matrix describing the relationship of the state covariance between two consecutive IMU measurements, and $Q_{i}$ is the noise covariance matrix created during the process of IMU measurements.

Using the covariance matrix ${\hat{P}}_{k}$ of the predicted state, the prior probability distribution of ${\tilde{x}}_{k}$ can be represented as follows.

(17) $$\begin{array}{c} {\tilde{x}}_{k}∼N\left(0,{\hat{P}}_{k}\right)\; \end{array}$$

#### 3.5.3. Iterated ESKF Optimization by Lidar Measurements

Based on the point-to-plane error computed by Equation (11) in Section 3.3.3, the lidar measurements can provide the posterior probability distribution. Let $h_{i}\left(x_{k}\right)$ denote the measurement model of the ith lidar point for the given state $x_{k}$. It is linearized at ${\hat{x}}_{k}$ using the method of first-order Taylor expansion. $H_{i}$ is the Jacobian matrix with respect to the error state ${\tilde{x}}_{k}$, and $v_{i}$ is created by the raw noise of lidar measurements. It can be represented as the posterior probability distribution at 0 with the covariance $R_{i}$.

(18) $$\begin{array}{cc} h_{i}\left(x_{k}\right)=h_{i}\left({\hat{x}}_{k}⊞{\tilde{x}}_{k}\right)= & h_{i}\left({\hat{x}}_{k}\right)+H_{i}{\tilde{x}}_{k}+v_{i}=E\left(p_{i},s_{i}\right)+H_{i}{\tilde{x}}_{k}+v_{i}=0\\ \; & v_{i}∼N\left(0,R_{i}\right)=-E\left(p_{i},s_{i}\right)-H_{i}{\tilde{x}}_{k} \end{array}$$

The ESKF update was performed iteratively to minimize the cost function represented as the maximum a posteriori estimation of the error state ${\tilde{x}}_{k}$; it is composed of the prior probability distribution of ${\tilde{x}}_{k}$ computed by IMU propagation and the posterior probability distribution of $v_{i}$ led by lidar measurements. The optimizing problem can be represented as below. *n* is the number of lidar points in the kth lidar scan and H is the Jacobian matrix of $\left({\hat{x}}_{k}⊞{\tilde{x}}_{k}\right)⊟{\hat{x}}_{k_{initial}}$ with respect to ${\tilde{x}}_{k}$. ${\hat{x}}_{k_{initial}}$ is the initial value of ${\hat{x}}_{k}$ taken from IMU propagation.

(19) $$\begin{array}{cc} \; & \underset{{\tilde{x}}_{k}}{min}\left({∥{\tilde{x}}_{k}∥}_{{\hat{P}}_{k}}^{2}+\sum_{i=1}^{n}{∥v_{i}∥}_{R_{i}}^{2}\right)=\\ \; & \underset{{\tilde{x}}_{k}}{min}\left({∥{\hat{x}}_{k}⊟{\hat{x}}_{k_{initial}}⊞H{\tilde{x}}_{k}∥}_{{\hat{P}}_{k}}^{2}+\sum_{i=1}^{n}{∥E\left(p_{i},s_{i}\right)+H_{i}{\tilde{x}}_{k}∥}_{R_{i}}^{2}\right) \end{array}$$

The problem is solved by computing the Kalman gain *K* and ${\hat{x}}_{k}$ iteratively. *H*, *R* and $E_{k}$ are composed of $H_{i}$, $R_{i}$ and $E\left(p_{i},s_{i}\right)$, respectively.

(20) $$\begin{array}{c} K={\left(H^{T}R^{-1}H+{\left(H^{-1}\;{\hat{P}}_{k}H^{-T}\right)}^{-1}\;\right)}^{-1}H^{T}R^{-1}\\ {\hat{x}}_{k}={\hat{x}}_{k}⊞\left(-KE_{k}-\left(I-KH\right)H^{-1}{(\hat{x}}_{k}⊟{\hat{x}}_{k_{initial}}\right)) \end{array}$$

The optimized state vector ${\hat{x}}_{k_{final}}$ is given as the final output of the odometry system until ${\hat{x}}_{k}$ converges to a certain small range or the number of iterations reaches a maximal threshold.

## 4. Experiments

The performance of the lidar-inertial odometry system proposed in this paper was tested mainly in terms of accuracy and robustness through a series of sufficient experiments. The effect on the system that the ground condition perception and optimization algorithm causes was analyzed in these experiments. We implemented the odometry in ROS kinetic 16.04 with a normal laptop with 8 GB RAM and Intel i5-9300 CPU. As the paper is focused on UGVs’ state estimation, the datasets used to evaluate the performance were gathered by the sensor-suites equipped on UGV. There are two sources of these datasets, including the open-source dataset used in the experiments of LIO-SAM [23] and LVI-SAM [34], and the dataset gathered by ourselves. The state-of-art lidar-inertial odometry systems used as comparisons were LINS [17], LIO-SAM, Lego-LOAM [25] and FAST-LIO2 [21]; these LIO systems were chosen to cover different types of lidar-inertial fusion methods. LINS is the representative work of tightly coupled lidar-inertial fusion methods using Kalman filter and feature-based method of lidar points registration, LIO-SAM is the representative work of tightly coupled methods using factor graph, Lego-LOAM is the representative work of loosely coupled methods and FAST-LIO2 is the representative work leveraging Kalman filter and direct method of lidar points registration.

### 4.1. System Performance in Open Dataset

In order to validate the effect of our ground condition perception and optimization algorithm on the precision of lidar-inertial odometry, a series of experiments were performed based on the two park datasets used in LIO-SAM and LVI-SAM. These datasets were gathered by driving a Clearpath UGV equipped with a sensor-suite composed of a Velodyne 16-line lidar, a MicroStrain 3DM-GX5-25 IMU and a Reach MGPS in unstructured areas. The dataset in LIO-SAM is small scale, covering approximately 0.7 km forested hiking trail, whereas the dataset in LVI-SAM is large scale, covering a journey of 4.6 km; there are various road conditions, including asphalt, grassland and dirt-covered trails in this dataset. Both datasets are challenging to LIO systems as the environments comprise changeable environmental elements and road conditions without prior knowledge.

#### 4.1.1. Visualization of the Ground Perception and Optimization Algorithm

**Figure 4:**
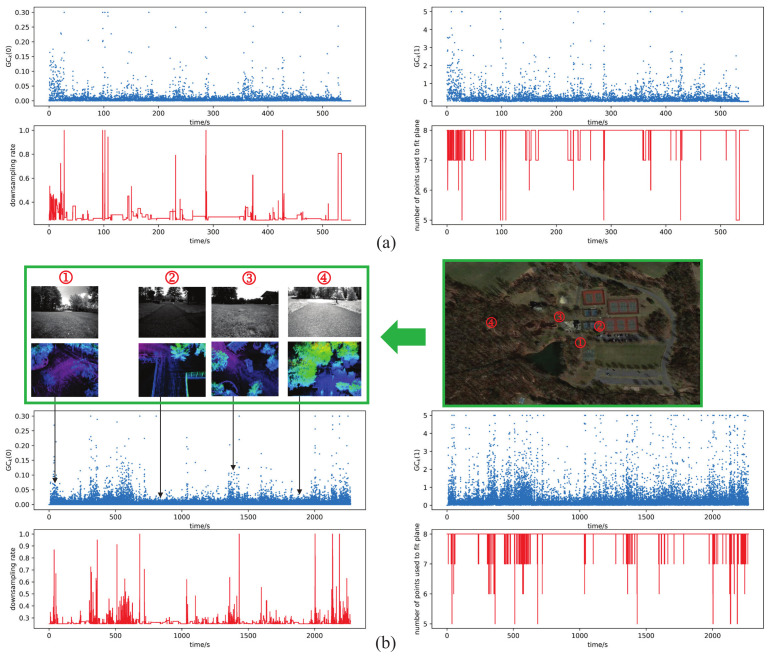
The changing process of the ground condition vectors, the downsampling rate of lidar points and the number of map points used to fit plane. (**a**) The situation in small-scale dataset of 500 s, 0.7 km. (**b**) The situation in large-scale dataset of 2200 s, 4.6 km. The pictures of ground are shown on the upper left of (**b**) as the visual information is included in the large-scale dataset, and the corresponding lidar point cloud maps are also provided below these pictures to reflect the ground conditions in detail. The picture on the upper right of (**b**) shows the locations of the ground condition in Google satellite map.

In Figure 4, in order to visualize the process of ground perception and optimization, the ground condition vectors of the two datasets are presented according to the perception method in Section 3.2; furthermore, the changing process of the downsampling rate of lidar points and the number of map points used to fit plane, which were introduced in Section 3.3, are also shown in this figure. The flatness of and changes in the ground can be described quantitatively by the ${GC}_{k}\left(0\right)$ and ${GC}_{k}\left(1\right)$ in the ground condition vector, respectively, the downsampling rate and the number of map points used to fit plane were adjusted as ${GC}_{k}\left(0\right)$ varies and ${GC}_{k}\left(1\right)$ was utilized as the activating signal of the optimization. We can see from (b) in Figure 4 that the grassland corresponds to bigger value of ${GC}_{k}\left(0\right)$ than that of the asphalt pavement, due to the grassland being more rough. As a consequence, the downsampling rate in the grass land was adjusted to be bigger than that in the asphalt pavement to save more details of the ground, and the number of map points used to fit plane was adjusted to be lower than that in the asphalt pavement to avoid incorrectly fitted plane and inconsistent point-to-plane error computation.

#### 4.1.2. Ablation and Comparison Study of the Ground Perception and Optimization Algorithm

**Figure 5:**
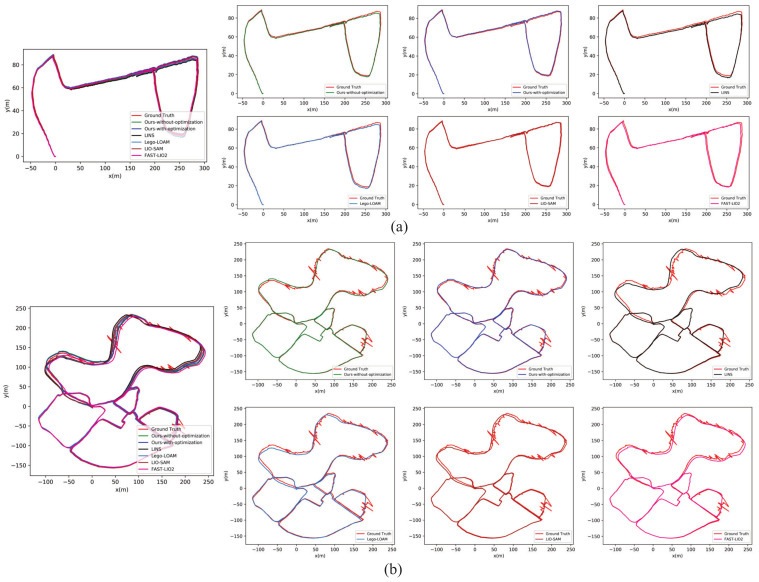
Trajectories of the datasets estimated by different LIO systems. (**a**) The trajectories evaluated in small-scale dataset. (**b**) The trajectories evaluated in large-scale dataset. In the small-scale dataset, GPS ground truth is available everywhere, whereas, in the large-scale dataset, GPS signal is partial available because of the blocking of forests. The ground truth will drift considerably in GPS-denied area.

Figure 5 shows the trajectories of the datasets evaluated by different methods. We disabled the ground condition optimization algorithm and used consistent downsampling rate of lidar points and unchanged number of map points used to fit plane to see the extra increase in accuracy that the algorithm brings to the LIO system. In addition, the trajectories evaluated by Lego-LOAM, LIO-SAM, LINS and FAST-LIO2 are also included as comparative result. In order to cope with the influence of loop closure and obtain consistent experimental results, we disabled the mechanism of loop closure in these methods. Table 1 shows the overall translational errors represented as the translational root means square error (RMSE) compared with GPS ground truth, and the regions with poor GPS signals were not included during the computation of RMSE. Due to the large-scale dataset starting and ending at the same position, the end-to-end translational and rotational errors were also considered as indices to evaluate the accuracy. It can be noticed that our method without ground optimization shows similar accuracy with LINS and FAST-LIO2 in both datasets; this is due to the fact that all three methods were adopted from iterated Kalman filter. The ground perception and optimization algorithm can significantly improve the accuracy of our LIO system according to the experimental results. In the small-scale dataset, where the ground condition does not change a lot, our system with the ground optimization could achieve similar accuracy of state estimation with LIO-SAM, and the translational RMSE with optimization was approximately 19% lower than the index achieved by the method without optimization, whereas, in the large-scale dataset, where the ground condition changes frequently, the extra enhancement in the ground optimization is more obvious: the RMSE with optimization is 26% lower than the method without optimization, the end-to-end translational error is reduced by 20% and the rotational error is reduced by 40% compared with the method without optimization. Furthermore, our complete LIO system could achieve the best accuracy compared with the other 4 state-of-art LIO systems in the large-scale dataset.

### 4.2. System Performance in Self-Gathered Dataset with Changing Ground Conditions

The accuracy and robustness of state estimation of the proposed LIO system were also tested in our self-gathered dataset. The sensor suite has a low cost, consisting of a RoboSense-16 lidar and a LMPS IMU, and the UGV carrying the sensors is a wheeled robot with Ackerman steering structure. The dataset was collected in an agricultural demonstration park, where we controlled the UGV to cross various ground conditions and scenes in the park, including the sidewalk composed of bricks, the bumpy grassland and the road made of asphalt. The travelling distance in each ground condition was approximately similar (approximately 500 m), which makes the dataset suitable for the usage of comparative experiments.

**Figure 6:**
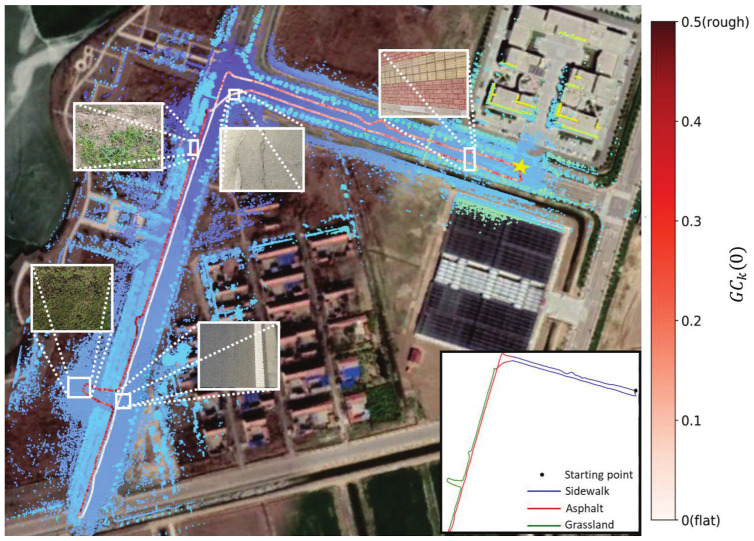
Trajectories and Point cloud map aligned with Google Earth satellite map of the park dataset. The dataset starts and ends at the same position which is indicated by the yellow star in the figure. The enlarged drawing is the real picture of the related ground condition. The detailed distributions of different types of ground conditions can be seen on the lower right corner.

Figure 6 shows the trajectories and the point cloud map produced during the process of UGV traveling in the park aligned with the satellite map in Google Earth using our complete LIO system. First, the pose of the starting point (shown as the yellow star) during the trajectory was recorded using real-time kinematic global positioning system (RTK-GPS), where the starting pose consists of the position (longitude, latitude, altitude) and the angle (roll, pitch, yaw) in WGS-84 (World Geodetic System 1984). Second, the position and the angle of the starting pose were transformed into universal transverse mercator (UTM) grid system) to obtain the transformation matrix. The trajectories and the point cloud map produced by our LIO system can be transformed into UTM using the transformation matrix. Finally, the trajectories and the point cloud map could obtain their positions (longitude, latitude, altitude) in WGS-84 using the inherent transformation between UTM and WGS-84; then, they could be aligned with Google satellite map. The flatness of the ground is also shown through the color of the trajectory in the figure. As the condition of ground varies from flat to rough, the color of the trajectory varies from white to red, representing ${GC}_{k}\left(0\right)$, which varies from 0 to 0.5. We can see from the figure that the ground condition of the grassland is the roughest, which implies that the larger downsampling rate and the smaller number of map points used to fit plane are needed to advance the accuracy of state estimation. This situation is opposite in asphalt, where the ground is flat. As a result, the large-scale point cloud map is well-matched with the satellite map, which means that the robustness and accuracy of state estimation in long term can be guaranteed; furthermore, the capacity of our system to adapt to the changing ground conditions is also reflected.

**Figure 7:**
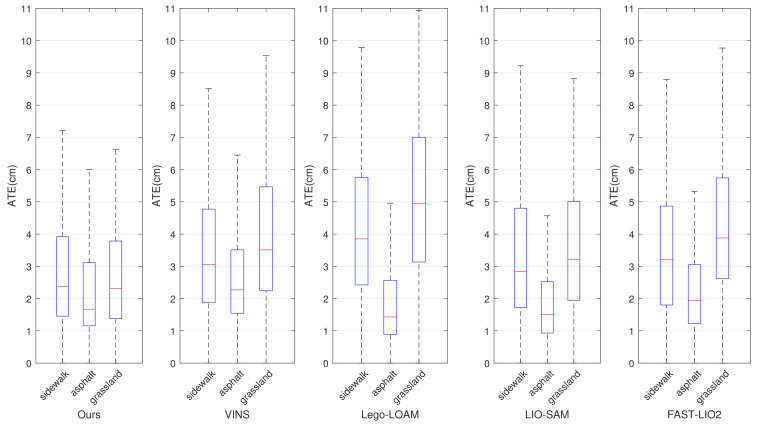
Absolute trajectory errors of the trajectories estimated by different LIO systems compared with GPS ground truth in different ground conditions.

The accuracy of state estimation of different methods in the self-gathered dataset is shown in Figure 7. It is represented as the absolute trajectory error (ATE)) of the trajectory sequence with respect to GPS ground truth. In order to show the robustness and consistent accuracy of the proposed method, the dataset was divided into three parts according to three different ground conditions (sidewalk, grassland and asphalt), and we measured the ATE separately in different ground conditions. The trajectory sequences produced by the LIO systems were firstly compared with the GPS ground truth, and then the sequences of the ATEs could be computed and obtained. Finally, the sequences of ATEs represented as arrays were imported into the box plot function of MATLAB to plot Figure 7. The figure produced by the box plot can display the distribution trend of the sample data, and can better visualize the ATE on the whole dataset and measure the robustness of LIO systems. It can be seen that the accuracy of the UGV’s states estimated by our method is similar in different ground conditions. Most of the ATEs fall into the interval from 1.5 cm to 2.5 cm, which means that the robustness under different ground conditions is guaranteed, whereas the other LIO systems cannot maintain the consistent accuracy of state estimation in changing ground conditions, and the ATEs in different ground conditions differ considerably for the comparative methods. The overall accuracy in the self-gathered dataset is shown in Table 2. Benefiting from the ground perception and optimization algorithm, our method shows a better overall accuracy than LINS and FAST-LIO2, which are also based on iterated ESKF. A similar overall accuracy with a little advantage can be achieved by our method compared with LIO-SAM, which is based on geometric feature-matching and factor-graph optimization, and a better accuracy is achieved by our system in rough ground conditions, such as a sidewalk and grassland. In the areas with flat ground conditions, Lego-LOAM performs the best accuracy as the plane hypothesis is made to improve the system performance on flat ground, but the overall accuracy in changing ground conditions cannot be guaranteed.

## 5. Conclusions

In this paper, based on the ESKF fusion model, the relationship between the flatness of the ground that the UGV is currently in contact with and the accuracy of the state estimation of the odometry was analyzed; then, in order to improve the accuracy and robustness of lidar-inertial odometry used by UGV traveling in various ground conditions, a ground condition perception and optimization algorithm along with the method to be embedded in the ESKF framework are proposed. The ground condition perception was realized by transforming the raw inertial information from IMU into a ground condition vector in real-time, and the optimization was realized by changing the parameters used in the lidar points processing module to adapt to the changing ground conditions. Through the results gained from the experiments in open datasets and self-gathered datasets with different ground conditions, the ground perception and optimization algorithm can enhance the performance of the LIO system by reducing approximately 20% of the translational RMSE, and the better accuracy and robustness of our system compared with several state-of-art LIO systems boosted by different algorithms are shown. The translational RMSE can achieve around 2%, which is enough to guarantee the accurate localization of a UGV in unstructured environments with various ground conditions. Furthermore, the capacity to maintain the consistency of the state estimation in changing ground conditions is also a characteristic of our method, and most of the ATEs of state estimation can stay at approximately 2 cm regardless of the ground conditions changing. However, our method is designed for ground vehicles and cannot be migrated to aerial vehicles, as the aerial vehicles do not contact the ground directly. In future work, visual information may be added in the ground perception and optimization algorithm to evaluate the flatness of the ground quantitatively and to broaden the application scenarios. 

## Figures and Tables

**Table 1:** Accuracy evaluation of the trajectories estimated by different methods and LIO systems in open dataset.

Small-Scale Dataset						
	**Our Method without Optimization**	**Our Method with Optimization**	**LINS**	**Lego-LOAM**	**LIO-SAM**	**FAST-LIO2**
**Estimated length (m)**	661.76	661.32	661.08	661.25	661.11	661.53
**Translational RMSE (%)**	1.49	1.21	2.34	1.57	1.12	1.52
**Large-Scale Dataset**						
	**Our Method without Optimization**	**Our Method with Optimization**	**LINS**	**Lego-LOAM**	**LIO-SAM**	**FAST-LIO2**
**Estimated length (m)**	4668.22	4670.55	4668.96	4677.23	4670.73	4667.81
**Translational RMSE (%)**	3.78	2.80	3.95	8.96	3.54	3.87
**End-to-end translational error (m)**	5.25	4.22	7.37	10.44	5.48	6.54
**End-to-end rotational error (m)**	3.43	2.07	4.80	5.17	2.18	3.66

The red text represents the optimal value of the related item.

**Table 2:** Accuracy evaluation of the trajectories estimated by different methods and LIO systems in self-gathered dataset.

Self-Gathered Dataset					
	**Our Method**	**LINS**	**Lego-LOAM**	**LIO-SAM**	**FAST-LIO2**
**Estimated length (m)**	1517.68	1518.24	1518.33	1517.14	1517.96
**Translational RMSE (%)**	2.60	3.61	3.93	2.85	3.22
**End-to-end translational error (m)**	3.21	3.94	4.73	4.28	4.01
**End-to-end rotational error (m)**	2.19	3.87	3.32	2.27	2.56

The red text represents the optimal value of the related item.

## Data Availability

Not applicable.

## Abbreviations

The following abbreviations are used in this manuscript:

UGV	Unmanned Ground Vehicle
ESKF	Error-State Kalman Filter
IMU	Inertial Measurement Unit
LIO	Lidar-Inertial Odometry
NDT	Normal Distributions Transform
LINS	Lidar-Inertial State Estimator
Lego-LOAM	Lightweight and Ground-Optimized Lidar Odometry and Mapping
LIO-SAM	Lidar-Inertial Odometry via Smoothing and Mapping
RMSE	Root Means Square Error
ATE	Absolute Trajectory Error
